# Making the Case for Continuous Quality Improvement in Percutaneous Coronary Intervention

**DOI:** 10.1016/j.jscai.2026.105632

**Published:** 2026-08-20

**Authors:** Karan Vasireddi, Derek Antoku, Arnold H. Seto

**Affiliations:** a Department of Medicine, VA Long Beach Healthcare System, Long Beach, California; b Department of Medicine, Charles R. Drew University, Los Angeles, California

**Keywords:** contrast nephropathy, coronary artery disease, guidance, quality improvement, transradial access, ultrasound

Since the first percutaneous intervention (PCI) was performed in 1977, numerous advancements have been made in the field of interventional cardiology. Although some early innovations (ie, stents) were quickly accepted, as the field has matured, newer best practices have suffered from slower adoption. These include transradial access, ultrasound guidance for vascular access, and standardized protocols to reduce the risk of acute kidney injury (AKI). Radial access has been shown to reduce bleeding complications and increase the rate of same-day discharge (SDD),^1^ which reduces healthcare costs and improves patient satisfaction.^2^^,^^3^ Ultrasound guidance reduces time to access and the rate of vascular complications.^4^ Lower contrast volumes are well-validated in reducing the incidence of postcatheterization AKI.^5^ Despite conclusive evidence of improved patient outcomes, changing practice patterns in the cath lab remains difficult.

One tool to address this issue is continuous quality improvement (CQI). CQI is a systematic, data-driven framework used by organizations to incrementally enhance processes, safety, and performance. The foundation of CQI is that poor outcomes are a result of systemic issues rather than individual failures. CQI relies on recurring cycles of planning, testing, and evaluating changes, summarized as the Plan-Do-Study-Act cycle (Figure 1).^6^

**Figure 1:**
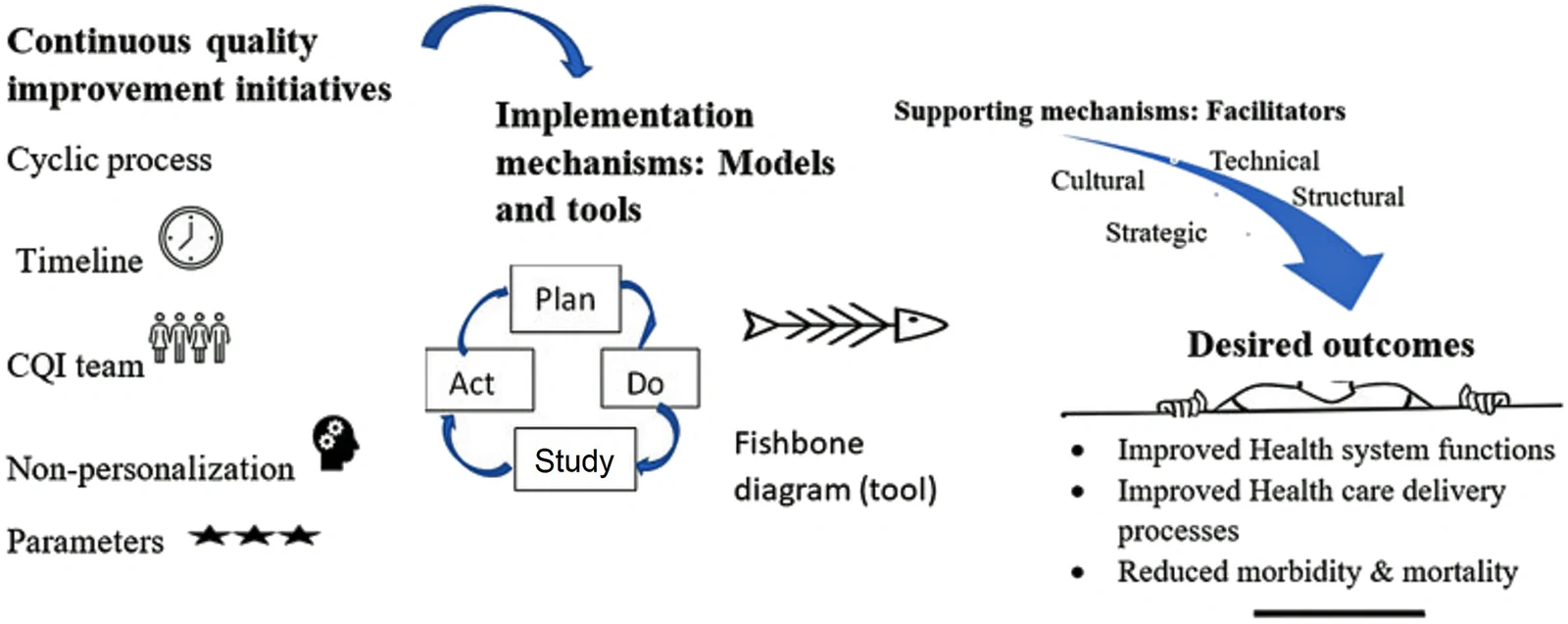
**Continuous Quality Improvement (CQI).** Adapted from Endalamaw et al.^6^

In this issue of *JSCAI*, Singla et al^7^ reported a single-center, retrospective cohort study of 2759 patients examining the outcomes of AKI, periprocedural hemoglobin drop, and SDD before and after establishing a CQI. Their CQI focused on several key interventions: (1) universal ultrasound guidance with a “radial-first” access protocol, (2) standardized estimated glomerular filtration rate–based hydration protocols and contrast limits, and (3) establishment of clear criteria to identify patients for SDD.

The major findings after implementation included a 43% increase in radial access, a 17% decrease in postprocedure bleeding, a 32% drop in postprocedure AKI, and a 47% increase in SDD. These improvements occurred despite a demonstrably sicker post-CQI cohort with increased rates of frailty (13.5% from 2.0%), heart failure (35.9% from 23.7%), and mechanical circulatory support use (3.9% from 1.1%), whereas in-hospital mortality remained unchanged (2.0% vs 1.5%; *P* = .32). The institution achieved these findings while increasing their procedure volume by 36%.

The study design had several strengths lending credence to the authors’ findings. Improvement in all 3 primary outcomes (AKI, reduction in postprocedure hemoglobin drop, SDD) survived Holm–Bonferroni correction for multiple outcome testing; the finding of reduction in AKI was further supported by multivariable logistic regression and a sensitivity model adjusting for confounders, strengthening internal validity. The study design also incorporated segmented regression and interrupted time series analyses, which helped separate a true change in trajectory at CQI implementation from pre-existing temporal trends, strengthening the causal interpretation of the volume, radial access, and SDD improvement.

It is important to acknowledge several limitations. First, this was a retrospective observational study and cannot prove causation. Second, the CQI program consisted of multiple interventions, making it difficult to pinpoint which specific changes were responsible for the improvement in outcomes. Third, it is unclear why there was a higher post-CQI transfusion rate (4.0 vs 2.2%; *P* < .001) despite a decrease in postprocedure hemoglobin drop; a sicker post-CQI population may have introduced selection bias with an increased incidence of heart failure and use of mechanical circulatory support. Another limitation is the lack of post-PCI renal function measurements in both cohorts, which is common with SDD. Lastly, the pre-CQI observation window spanned the COVID-19 pandemic when SDD became the default for many facilities and encompassed a period in which there was already a national trend toward increased use of radial access and contrast reduction.

Singla et al^8^ have shown that CQI programs can be an effective tool for improving patient outcomes in the cardiac cath lab. Quality efforts elsewhere have demonstrated similar benefits. The Blue Cross Blue Shield of Michigan Cardiovascular Consortium has successfully increased rates of intracoronary imaging during PCI to 25% through a pay-for-performance program. ChristianaCare in Delaware successfully implemented closed-loop communication to reduce medication and equipment errors.^9^

The cath lab can be a challenging environment for quality improvement due to variability in patients, presentations, operators, and staff, but such challenges are not insurmountable and cannot be excuses for inaction when we know what works to improve patient outcomes and safety. We applaud the authors for demonstrating that CQI made their cath lab safer and hope more follow their example.

## CRediT authorship contribution statement

**Karan Vasireddi:** Writing – original draft, Writing – review & editing. **Derek Antoku:** Writing – review & editing. **Arnold H. Seto:** Conceptualization, Writing – review & editing.

## Declaration of competing interest

Arnold H. Seto is a speaker for General Electric, Getinge, and Gore, and is a consultant for SummaCor, Merit Medical, and Terumo. Karan Vasireddi and Derek Antoku reported no financial interests.
